# The impact of COVID-19 on multidrug-resistant organisms causing healthcare-associated infections: a narrative review

**DOI:** 10.1093/jacamr/dlac130

**Published:** 2022-12-29

**Authors:** Lucy S Witt, Jessica R Howard-Anderson, Jesse T Jacob, Lindsey B Gottlieb

**Affiliations:** Division of Infection Diseases, Emory University School of Medicine, Atlanta, GA, USA; Division of Infection Diseases, Emory University School of Medicine, Atlanta, GA, USA; Emory Antibiotic Resistance Group, Emory University, Atlanta, GA, USA; Division of Infection Diseases, Emory University School of Medicine, Atlanta, GA, USA; Emory Antibiotic Resistance Group, Emory University, Atlanta, GA, USA; Division of Infection Diseases, Emory University School of Medicine, Atlanta, GA, USA; Emory Antibiotic Resistance Group, Emory University, Atlanta, GA, USA

## Abstract

Coronavirus disease 2019 (COVID-19) changed healthcare across the world. With this change came an increase in healthcare-associated infections (HAIs) and a concerning concurrent proliferation of MDR organisms (MDROs). In this narrative review, we describe the impact of COVID-19 on HAIs and MDROs, describe potential causes of these changes, and discuss future directions to combat the observed rise in rates of HAIs and MDRO infections.

## Introduction

According to the US CDC, 1 in 31 hospitalized patients develops at least one healthcare-associated infection (HAI) each day.^1^ HAIs of particular concern include central-line associated bloodstream infections (CLABSIs), catheter-associated urinary tract infections (CAUTIs), ventilator-associated pneumonias (VAPs) and surgical site infections (SSIs).^2^ HAIs cause significant morbidity and mortality for patients, and they also directly impact hospitals’ financial health as pay for performance metrics. Coronavirus disease 2019 (COVID-19) has changed healthcare delivery as a whole, and infection prevention in particular, in a myriad of ways. Early in the pandemic, many hospitals suffered a shortage of personal protective equipment (PPE) and had to halt or revise standard infection prevention duties to conserve scarce human and equipment resources and to focus on safely caring for patients with this novel pathogen. However, COVID-19 also ushered in changes that had the potential to reduce HAI rates, including heightened awareness of the risk of pathogen transmission, emphasis on hand hygiene, and universal masking. Later in the pandemic, burnout and staffing instability adversely impacted the ability to deliver consistent, reliable care, reversing a decade of reduction in the rates of HAIs. In this narrative review we will describe the impact of COVID-19 on the frequency of HAIs globally, with a focus on MDR organisms (MDROs). We will outline potential causes of these trends and describe potential future initiatives to reduce the occurrence of MDRO HAIs.

## Methods

In collaboration with a library informationist, we queried PubMed for articles describing HAIs during the COVID-19 pandemic. A comprehensive literature review was completed for articles investigating MDROs in relation to COVID-19, which yielded 217 results, all of which were reviewed for inclusion. Please see the Supplementary data at *JAC-AMR* Online for the full search term. We also reviewed references from included manuscripts to identify other relevant studies.

## Results

### Global trends

#### Healthcare-associated infections

HAIs have increased with COVID-19 worldwide.^3–12^ This change was most notable for CLABSIs, which increased in Europe^11,13,14^ and the USA in 2020 and 2021,^7–10,15–17^ with a particularly concerning rise in candidaemia.^18–22^ In the USA, this rise reversed almost 5 years of reduction in rates of CLABSI.^23^ The rise in HAIs appeared to be proportional to the number of COVID-19 cases.^15,16^ Germany, where only 5% of the country was infected with COVID-19 in early 2020, did not see an increase in HAIs during the first months of the pandemic.^24^ In contrast, countries such as the USA, where an estimated 13% of the population had COVID-19 in early 2020, noted a concurrent rise in HAIs.^24^ Similarly, a retrospective study of CLABSIs in the USA found that in hospitals where >10% of admitted patients had COVID-19 there were significantly higher rates of CLABSIs compared with hospitals where COVID-19 accounted for <5% of admissions.^25^ VAPs increased across Europe^26–28^ and North^8,9,17^ and Latin America.^6^ Conversely, in the USA CAUTIs increased only slightly in 2020 and 2021^1,17^ while *Clostridioides difficile* infections decreased^8,17,29^ and SSIs remained stable.^17^

#### MDROs in HAIs

Since the start of the COVID-19 pandemic there has been an increase in many MDROs including carbapenem-resistant *Acinetobacter baumannii* (CRAB), antifungal-resistant *Candida*, ESBL-producing Enterobacterales and VRE.^30^ Conversely, the overall rates of some infections including carbapenem-resistant Enterobacterales (CRE), MDR *Pseudomonas* and MRSA remained stable. However, the rates of most *hospital-onset* infections increased in both 2020 and 2021.^17^ The CDC reported a 78% increase in hospital-onset CRAB, 35% increase in hospital-onset CRE, 32% increase in hospital-onset ESBL-producing Enterobacterales, 13% increase in hospital-onset MRSA and a 14% increase in hospital-onset VRE.^30^ This same trend was reflected around the world: endotracheal aspirates obtained from patients in a Turkish ICU showed an increase in MDROs after March 2020 as compared with the year prior,^31^ a national referral hospital in Indonesia noted an increase in MDRO bloodstream infections from 130.1 cases per 100 000 patient-days in 2019 to 165.5 cases per 100 000 patient-days in 2020 (incidence rate ratio 1.016 per month, *P* value <0.001),^32^ an ICU in Rome saw a statistically significant association between COVID-19 diagnosis and drug-resistant *A. baumannii* bloodstream infections^33^ and France noted an increase in resistance to third-generation cephalosporins among *Klebsiella*, Enterobacteriaceae and *Pseudomonas aeruginosa* bloodstream infections in 2020 compared with 2019.^34^

### MDRO outbreaks

There have been increased reports of outbreaks of HAIs due to MDROs. Although rates of MRSA increased in many locations^8^ and Gram-positive outbreaks were reported in Europe,^35–37^ the majority of COVID-19-associated MDRO outbreaks consisted of Gram-negative organisms. MDR Gram-negative outbreaks (of both infections and colonization) were reported in Mexico,^38^ Spain,^39^ France,^40^ the USA^41^ and Iran.^42^ The majority of outbreaks occurred in ICUs. Ghanizadeh *et al*.^42^ found distinct genetic clusters of *Klebsiella pneumoniae* in tracheal secretions of 70 patients with COVID-19 infections requiring mechanical ventilation in an Iranian ICU, suggestive of many smaller outbreaks within their ward. In a French ICU dedicated to caring for patients with COVID-19, they noted an outbreak of CTX-M-producing *K. pneumoniae*, including 16 infections between March and June 2020. This same ICU also noted an increase in rates of CRE compared with pre-pandemic levels.^40^ Some outbreaks were hospitalwide: during the initial months of the pandemic, a Peruvian hospital experienced an outbreak of NDM-producing *K. pneumoniae* among COVID-19 patients, a pathogen the hospital had never previously identified.^43^ Similarly, a New York City medical centre identified five patients with COVID-19 who developed secondary infections including bloodstream infections and VAPs from NDM-producing *Enterobacter cloacae* between March and April 2020, representing a previously unseen cluster of infections.^41^

CRAB outbreaks were frequently reported. A cluster of 34 CRAB cases were identified between February and July 2020 at a single acute care hospital in New Jersey during a surge of COVID-19 admissions and resolved after returning to usual infection prevention practices.^44^ Similar outbreaks and increases in infections were noted in other US,^45^ Swiss^46^ and Brazilian ICUs,^47–49^ including a Brazilian outbreak mostly driven by International Clone 2, an unusual cause of CRAB in Latin America.^49^ Eckardt *et al*.^45^ described a cluster of six CRAB cases (five infections) in their COVID-19-dedicated ICU in Florida in late 2020 where no prior CRAB had been detected. Thoma *et al*.^46^ described two CRAB outbreaks in their Swiss medical and surgical ICUs in late 2020, detected in both clinical and screening isolates. Using WGS, they identified the index patients for both outbreaks, both of whom had been transferred from the Balkans. Although transfers from this region were common prior to 2020, CRAB outbreaks were not, suggesting that factors associated with the COVID-19 pandemic directly contributed to the spread of this MDRO. Outbreaks were noted in both non-ICU and ICU patients with COVID-19 in Italy and Israel.^33,50^ The outbreak described in an Israeli hospital, where CRAB is endemic, occurred despite extensive terminal cleaning measures and was postulated to be due to persistent environmental contamination.^50^

Multiple reports of *Candida auris* outbreaks emerged, including in a COVID-19 unit in Florida,^51^ as well as in Mexico,^52^ Italy^53,54^ and Lebanon^55^ (the first described in this country) and in an ICU in India.^56^ These outbreaks were identified from both clinical and screening isolates, and they occurred in patients with and without COVID-19, and in both ICUs and hospital wards. A rise in the rate of fluconazole-resistant *Candida parapsilosis* was noted in Europe.^22,57^ Outbreaks of this organism were also described in two connected hospitals in Brazil,^58^ likely resulting from a shared, contaminated CT scanner. Outbreaks were reported in Spain,^59^ and in Turkey, where the acceleration of an on-going resistant *C. parapsilosis* outbreak, present since 2015, was confirmed using WGS.^60^

### Factors underlying trends in HAIs and MDROs

**Figure 1.**
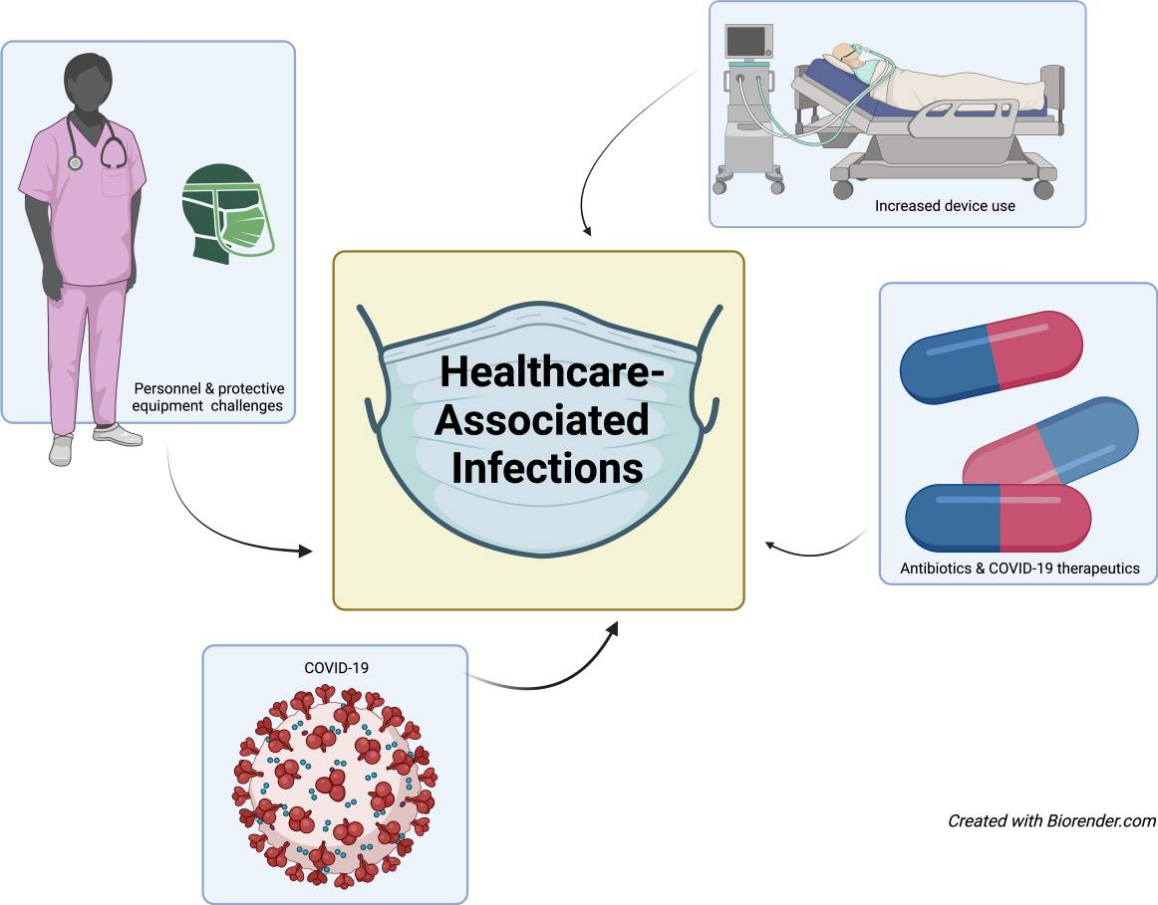
Factors contributing to HAIs are interconnected. COVID-19 sickened staff and caused burnout, reducing the number available to care for ill patients. Increased critically ill patients altered nurse-to-patient ratios, stretching personnel thinly. Critically ill patients required COVID-19-directed therapeutics that predisposed patients to infections. PPE was meant to protect staff and reduce infections but scarcity led to suboptimal utilization and disease transmission.

Factors contributing to the rise in HAIs and MDROs are varied but interrelated and include human factors such as staffing instability, delayed care and increased device use. Similarly, COVID-19 infection itself, therapeutics used to treat patients with COVID-19, along with increased and often inappropriate antibiotic use likely also contributed (Figure 1).

#### COVID-19 as a risk factor

COVID-19 causes intense multisystem inflammation responsible for most of the morbidity and mortality associated with infection. Local immune dysregulation likely contributes to the observed increase in HAIs in those infected with COVID-19. COVID-19 pneumonia is thought to increase the risk of VAPs via associated atelectasis, microemboli, pulmonary infarction and lymphopenia.^61^ COVID-19 has also been associated with a decrease in pulmonary microbiome diversity, making the lung more susceptible to opportunistic, hospital-acquired pathogens.^62^ While many centres reported surges in HAIs and MDROs, an Italian surgical, *non-COVID* ICU noted a decrease in HAIs during the pandemic compared with years prior, with a specific decrease in MDR pathogens, suggesting that the COVID-19 infection itself may be a risk factor for in-hospital acquisition of infection.^63^

#### Human factors and equipment as a risk factor

As repetitive waves of COVID-19 infections pummelled communities, ill patients inundated hospitals. This influx of patients led to multiple shortages in equipment and staff and strained the resilience of healthcare personnel (HCP).^64^ Staff shortages due to burnout or illness led to higher patient-to-nurse ratios, along with increases in the absolute number of patients cared for in a given unit or facility, more frequent staff turnover, and reassigning of staff to unfamiliar units or positions.^6,40,46,47,49^ HCP described confusion regarding the most up-to-date guidance regarding PPE, lack of interest in infection prevention, increase in workload, and decreased time to care for patients due to staff shortages.^15,65^ Initially, PPE was scarce and efforts to preserve the limited supply while protecting HCP resulted in decreased patient contact as well as reuse and extended wear of available PPE.^39,41,45,59,66^ This focus on keeping HCP safe increased the risk of patient-to-patient MDRO transmission due to reuse of gowns and even gloves between patients on the same unit,^67^ as exemplified by an outbreak of hepatitis E between hospitalized patients in the UK.^68^ Use of indwelling devices including urinary catheters, central lines and ventilators increased, providing more opportunities for device-related infections.^69,70^ IV infusion pumps were kept outside of patient rooms to conserve PPE and minimize the number and duration of interactions between patients and HCP.^45,71^ Interventions proven to reduce HAIs such as adherence to central line and ventilator maintenance bundles were missed due to fewer or shorter interactions between patient and HCP, and competing priorities given the critical illness of these patients.^15,41^ Patient proning, thought to improve mortality in patients with COVID-19 and acute respiratory distress syndrome, made central line care challenging.^72^ In the USA, patient length of stay increased in 2020, providing more time for pathogen acquisition.^1,73,74^ Furthermore, infection prevention teams shifted their focus from reduction of HAIs to helping HCP safely care for patients with COVID-19.^41^

#### COVID-19 therapeutics as a risk factor

COVID-19 therapeutics increase the risk of HAIs.^41^ Dexamethasone was shown early in the pandemic to decrease mortality in patients who required oxygen therapy.^75^ Unfortunately, dexamethasone also has been shown to lower HLA-DR expression and CD4 cell circulation in COVID-19 patients,^76^ predisposing them to fungal and bacterial infections.^73^ Corticosteroid use was associated with increased bloodstream infections and increased the odds of death (OR 8.8, 95% CI 3.5%–22.1%) in an Italian ICU.^33^ Similarly, in a case series of hospitalized patients in Brazil, the authors noted that all 11 patients who developed candidaemia did so after receiving steroids and that this infection occurred at a rate 10 times higher than patients without COVID-19 during the same time period.^77^ Patients with COVID-19 who were treated with dexamethasone were more likely to be diagnosed with a VAP in a propensity-matched ICU cohort in Italy (HR 1.81, 95% CI 1.31–2.50)^78^ and France (adjusted HR of 1.29, 95% CI 1.05–1.58),^79^ and had an increased risk of MDRO infection in South Korea (adjusted OR 6.09, 95% CI 1.02–36.49).^80^

Tocilizumab, an anti-IL-6 medication, and baricitinib, a JAK kinase inhibitor, are used in patients with rapidly progressing COVID-19.^81,82^ Despite the clinical improvement these drugs can provide, they also increase the risk of bacterial infection, including HAIs. Patients with COVID-19 prospectively studied in an Italian ICU diagnosed with an additional coinfection were more likely to have received tocilizumab or baricitinib (OR 5.09, 95% CI 2.2–11.8).^83^ A similar result was noted in multiple retrospective observational cohorts in the USA.^73,84,85^ Tocilizumab was associated with an increased risk of *Candida* bloodstream infections, as described in a case–control study involving two Indian ICUs between August 2020 and January 2021 (adjusted OR 11.952, 95% CI 1.431–99.808).^86^ These findings are not surprising given both drugs’ effects on the immune system, and their previously described association with infection when used for rheumatological diseases.^87–91^

#### Antibiotic use and MDROs

High rates of antibiotic utilization in patients with COVID-19 contributed to an increase in HAIs caused by MDROs. Diagnostic uncertainty, limited evidence-based treatment options, severity of illness and initial concern for bacterial coinfection all drove increased antibiotic use.^92^ A cross-sectional survey of Scottish hospitals noted that over 60% of patients admitted for COVID-19 received antibiotics on any given day.^93^ A review of 126 COVID-19 patients requiring mechanical ventilation in three Seattle-area hospitals found that 97% received antibiotics, although only 55% were diagnosed with a VAP and 20% with a bloodstream infection.^94^ Early reports from China noted the majority of admitted patients received antibiotics,^95,96^ and a subsequent international meta-analysis found that 72% of patients admitted for COVID-19 globally received antibiotics.^97^ Furthermore, authors of a retrospective cohort of hospitals in South Carolina noted that when compared with the same months in 2019, hospitals that admitted patients with COVID-19 saw increased antibiotic usage between March and June 2020 (mean difference of 34.9 days of therapy/1000 days, 95% CI 4.3–65.6, *P* value 0.03). Most of the increased usage was in broad-spectrum antibiotics (defined as antipseudomonal penicillins, cephalosporins, carbapenems and aminoglycosides) with a 16.4% increase in agents used for hospital-acquired pathogens. Hospitals that did not admit patients with COVID-19 saw no change in their antibiotic utilization during these time periods.^98^ Increased antibiotic use during COVID-19 has also been described in Portugal,^99^ the Netherlands,^100^ elsewhere in the USA^41,101–103^ and Singapore,^104^ often with a specific increase in broad-spectrum antibiotics.^105^ This increase persisted despite a paucity of data regarding antibiotic efficacy^106^ and multiple studies showing that less than 7% of patients with COVID-19 had a concomitant bacterial infection upon admission.^100,107–110^

With increased antibiotic use and a shift in infection prevention practices to focus on COVID-19, came an increase in MDROs. A single-centre case–control study conducted in Spain between March and May 2020 found higher rates of CRE in patients admitted for COVID-19 compared with those admitted for other reasons (1.1% versus 0.5%, *P* value 0.005) with statistically significant higher rates of antibiotic usage (100% of patients with COVID compared with 75% of those without, *P* value 0.004), specifically increased ceftriaxone and carbapenem use.^69^ A hospital in Rome noted higher rates of MDROs in COVID-19 units than non-COVID-19 units (29% versus 19%, *P* value <0.05), which they attributed to broader use of antibiotics in the COVID-19 units.^111^ In a retrospective cohort of patients admitted for COVID-19 to a single hospital in New York between March and April 2020, they noted that 100% of the patients who developed a superinfection with an MDRO had received antibiotics in the prior 30 days compared with only 65% of those with an infection that was not caused by an MDRO.^112^ Those with an infection caused by an MDRO received antibiotics for a median of 12 days, compared with a median of 8 days for those with an infection caused by a susceptible organism.^112^ This hospital also noted a shift in their antibiogram between 2018 and 2020 with a greater than 10% decline in susceptibility of *K. pneumoniae* isolates to many β-lactams including cefepime, meropenem and piperacillin/tazobactam.^112^ Although antibiotic prescribing patterns have hopefully improved for patients with COVID-19, the sequelae of antibiotic overuse and increase in MDROs continue to plague the global healthcare system.

## Limitations

Although we have presented many studies reporting increased rates of HAIs, MDROs or MDRO outbreaks during the COVID-19 pandemic, some of these reports did not provide pre-pandemic data. The pandemic has likely led to heightened surveillance of MDROs and many of the healthcare systems in these studies may not have had robust tracking systems in place prior to COVID-19, and thus direct comparisons may be limited. The authors of many reports frequently postulate reasons for a rise in HAIs and MDROs, but few studies provide data behind their assertions. Still, the explanations offered are consistent, founded on known epidemiological and biological risk factors, and many authors describe successfully ending outbreaks by intervening in their proposed causal pathways. Furthermore, in the USA the Centers for Medicare and Medicaid (the nationally funded and largest healthcare payer in the country) allowed participating facilities to ease their reporting of HAIs, a metric previously required for reimbursable quality evaluations.^113^ Thus, rates of HAI reporting declined in 2020 and reports from this time may not wholly describe national trends in the USA. This exemption ended in the second quarter of 2020, and reporting rates for 2021 have returned to pre-pandemic levels.^17^

## Future directions

Infection prevention practices and antibiotic prescribing have stabilized since the beginning of the pandemic, with a renewed focus on combatting HAIs and MDROs. Hand hygiene was prioritized with the onset of COVID-19, which will hopefully persist as the pandemic’s urgency subsides.^114^ Enzymatic detergents and isopropyl alcohol, shown to decrease contamination from COVID-19, are also effective against some Gram-negative bacteria,^115^ and drug-resistant outbreaks have prompted the development of novel disinfecting agents and protocols to eradicate *C. auris* as well as other MDROs.^116,117^ Outbreaks of highly resistant organisms prompted usage of WGS to identify clusters and potential pathways of transmission.^54,60^ This approach is becoming more commonplace in infection prevention investigations as the availability of WGS increases and associated costs decrease, which may lead to faster, more accurate identification of MDRO outbreaks.^118,119^ Similarly, the rapid diagnostics employed for COVID-19 are comparable to many rapid diagnostics available to identify bacterial resistance. This similarity may encourage hospital authorities to invest in broader deployment of point-of-care resistance tests in clinical microbiology laboratories.^114^ COVID-19 has ushered in the acceptance of telehealth and opportunities for remote antibiotic stewardship and infection prevention consults in long-term care facilities and smaller community hospitals that were disproportionately affected by a rise in HAIs during COVID-19.^12^ There has also been renewed focus on antimicrobial stewardship itself, specifically with regard to patients with COVID-19.^30,92^ Lastly, novel antibiotics continue to be developed to treat the most drug-resistant bacteria.^120^

Nevertheless, challenges remain. Hospital staff shortages, which developed during the pandemic due to high turnover, early retirement, burnout and reliance on contract staffing, continue to plague hospitals across the world. The CDC recently recommended that masking requirements could be eased at some healthcare facilities, adding complexity and confusion within many communities.^121^ Conditions that encourage the appropriate use of PPE and decrease opportunities for infection must be hardwired through quality improvement projects and human factors research to control the rise of HAIs.

## Conclusions

The COVID-19 pandemic has permanently changed healthcare and at least temporarily increased the rates of HAIs and MDRO cases throughout the world. However, with this comes increased recognition of the importance of infection prevention and attention on antimicrobial stewardship. As the world becomes more accustomed to COVID-19, healthcare systems must refocus on preventing HAIs and MDRO infections.

## Supplementary Material

dlac130_Supplementary_Data

## Supplementary data

PubMed search term available as Supplementary data at *JAC-AMR* Online.
