# Supplementary material for: The impact of COVID-19 on multidrug-resistant organisms causing healthcare-associated infections: a narrative review
Source: JAC Antimicrob Resist. 2022 Dec 29;5(1):dlac130. doi: 10.1093/jacamr/dlac130 (PMC9798082; doi:10.1093/jacamr/dlac130)
Supplement: dlac130_Supplementary_Data [file dlac130_supplementary_data.docx]

**Title:** The impact of COVID-19 on multi-drug resistant organisms causing healthcare-associated infections: a narrative review – Supplement

**PubMed Search Term:**

((("cross infection"[MeSH Terms] OR "cross infection*"[Text Word] OR "healthcare associated infection*"[Text Word] OR "nosocomial infection*"[Text Word]) AND ("covid 19"[Text Word] OR "covid 19"[MeSH Terms] OR "sars cov 2"[Text Word] OR "sars cov 2"[MeSH Terms] OR "severe acute respiratory syndrome coronavirus 2"[Text Word] OR "ncov"[Text Word] OR "2019 ncov"[Text Word] OR ("coronavirus"[MeSH Terms] OR "coronavirus"[Text Word] OR "cov"[Text Word]))) NOT ("covid 19 vaccines"[Text Word] OR "covid 19 vaccines"[MeSH Terms] OR "covid 19 serotherapy"[Text Word] OR "covid 19 serotherapy"[Supplementary Concept] OR "covid 19 nucleic acid testing"[Text Word] OR "covid 19 nucleic acid testing"[MeSH Terms] OR "covid 19 serological testing"[Text Word] OR "covid 19 serological testing"[MeSH Terms] OR "covid 19 testing"[Text Word] OR "covid 19 testing"[MeSH Terms])) AND ("resist"[All Fields] OR "resistance"[All Fields] OR "resistances"[All Fields] OR "resistant"[All Fields] OR "resistants"[All Fields] OR "resisted"[All Fields] OR "resistence"[All Fields] OR "resistences"[All Fields] OR "resistent"[All Fields] OR "resistibility"[All Fields] OR "resisting"[All Fields] OR "resistive"[All Fields] OR "resistively"[All Fields] OR "resistivities"[All Fields] OR "resistivity"[All Fields] OR "resists"[All Fields])
